# Reducing blood wastage through introduction of a transfusion team

**DOI:** 10.1186/s13049-025-01452-w

**Published:** 2025-08-11

**Authors:** Biswadev Mitra, Ruby Garland, Jackson Catalano, Gerard O’Reilly, Alexandra Nevill

**Affiliations:** 1 School of Public Health & Preventive Medicine, Monash University, 553 St. Kilda Rd, Melbourne, VIC 3004 Australia; 2 Emergency & Trauma Centre, The Alfred Hospital, Melbourne, VIC Australia; 3 Royal Australian Air Force, No. 1 Expeditionary Health Squadron, Amberley, QLD Australia; 4 Pathology Department, The Alfred Hospital, Melbourne, VIC Australia

**Keywords:** Blood transfusion, Blood component transfusion, Death, Shock, hemorrhagic, Emergency medicine, Patient blood management

## Abstract

**Background:**

In the intense setting of reception and resuscitation of critically bleeding patients, wastage of up to 10% of blood components occur, commonly due to prolonged breaks in the cold chain. Additionally, some transfusions may be avoidable. The aim of this study was to assess the impact of dedicated transfusion teams (staff focused solely on blood handling during resuscitation) on blood component wastage and transfusion volumes.

**Methods:**

This was a retrospective pre- and post-intervention study. We introduced a transfusion team for all episodes of blood transfusion in a level 1 trauma centre. The core elements of the intervention were: (1) dedicated ‘blood checkers’ without other competing duties during the resuscitation, (2) a 17-minute timer activated on receipt of blood components, (3) telephone for communication with the blood bank, and (4) a pre-determined process of blood component request, usage and return discussed at the initial team huddle.

**Results:**

Patient demographics, indications for transfusion and massive transfusion rates were similar between the pre- and post-intervention periods. During 01 Jan 2019 to 30 Jun 2021 (pre-intervention), 109 (1.7%) of 6619 blood component units dispensed were wasted. Following the intervention, until 30 Jun 2023, 73 (1.1%) of 6575 units were wasted (*p* = 0.008). In the post intervention period, median patient transfusion volumes were significantly lower at 2 (inter-quartile range; IQR 2–6) compared to 3 (IQR 2–6) units (*p* = 0.002).

**Discussion:**

Dedicated transfusion teams during emergency use of blood were associated with lower wastage and lower transfusion volumes. Transfusion teams may lead to more precise management of critical bleeding, while enabling cognitive capacity for the team leader to focus on identifying and controlling haemorrhage.

## Introduction

Donated human blood is a precious resource with constant imbalance in supply and demand [1, 2]. In addition to donor inertia, the availability of blood components may be constrained by supply chains, particularly in austere environments [3]. The costs of blood components are substantial, and often consist of hidden aspects not commonly accounted for in simple economic terms [4]. The judicious use of blood and blood components, is therefore, an ethical and economic necessity.

The practice of emergency medicine requires simultaneous integration of several bodies of knowledge, skills, and behaviours, often under the pressure of time. The nature of treating multiple critically ill patients require clinicians to sustain their focus and mental effort for extended periods. The ability to avoid and mitigate the effects of cognitive overload of the team leader during resuscitation may be critical to patient outcomes [5]. 

In the hectic stages of reception and resuscitation of critically bleeding patients, avoidable transfusions have been commonly reported [6, 7]. The key determinants of avoidable transfusions are inaccurate estimations of blood loss and delays to recognition of control of critical bleeding. The detection and estimation of blood loss rely primarily on event and patient history, examination and vital signs, that are all poorly specific for critical bleeding. Similarly, physiological response to resuscitation and results of laboratory tests are often delayed, resulting in avoidable transfusions.

Another source of blood wastage are deviations from specified temperature ranges that can affect the viability of the constituents of blood. Following delivery of blood to the emergency department, transfusion may be delayed. Reasons for delay include prioritisation of other lifesaving interventions, inadequate intravenous access, fluctuating patient conditions and sometimes, lack of staff to initiate the transfusion. During this time, the blood components are in room temperature and not refrigerated. Prolonged breaks in the cold chain can lead to reduced effectiveness of the blood component, increase the risk of bacterial proliferation in blood components and may cause potentially life-threatening transfusion reactions [8]. This mandates the need to discard such blood units. For example, the Australian Red Cross Lifeblood mandates that any red cell components returned to the blood bank which have remained out of storage conditions of 2–6 °C for longer than 30 min must be disposed.

We hypothesised that a transfusion team, dedicated to the receipt of blood components and delivery, could reduce blood wastage and avoidable transfusions. The aim of this study was to assess the effect of introduction of a transfusion team and processes for emergency blood transfusion on blood component wastage and transfusion rates.

## Methods

### Design

This was a retrospective analysis pre- and post- an intervention, designed to improve transfusion practices in the emergency department. The pre-intervention period spanned from 01 Jan 2019 to 30 Jun 2021. The post-intervention period was from 01 July 2021 to 30 Jun 2023.

### Setting

The Alfred Hospital is an adult tertiary referral hospital in Melbourne, Australia, incorporating a level 1 trauma centre. It has an annual census of over 75,000 presentations.

### Intervention

There were four changes to previous processes. These changes were implemented at the same time and concurrently, commencing 01 July 2021. Dedicated staff were allocated to receive blood components from the blood bank and transfusion to patients. This team was responsible for receipt of blood from the blood bank, checking identifiers and crossmatching, confirming the prescription of blood, delivery of the blood components, and disposition blood bags, whether used or not. The intervention was deployed uniformly on all days of the week and at all hours. There were four key components of the intervention. (1) It was recommended that these staff would not perform other tasks during resuscitation and would not be disturbed during the reception of delivery of blood. (2) A timer was made available for every transfusion that was activated on receipt of a blood component and provided a sound stimulus after 17 min. This limit was arbitrarily chosen to allow sufficient for the blood to be returned to the blood bank and then into the cold chain without breach. (3) A dedicated phone was provided for communication with the blood bank. (4) The workflow of blood component reception and delivery was formalised using a flow chart and where relevant, discussed during resuscitation team time-out during the planning phase of the resuscitation, when possible.

### Outcomes

The primary outcome was the proportion of blood units that were wasted (had to be discarded due to prolonged breaks in the cold chain) with the number of blood units transfused as the denominator. The secondary outcome was the units of blood components transfused per patient.

### Statistical analysis

Baseline characteristics summarised using continuous variables were compared using Student’s t-test and categorical variables were compared using the chi-square test. The proportion of blood components wasted was compared using the chi-square test. To further explore the pattern of change in blood wastage, we performed an interrupted time-series analysis using ordinary least- square regression. Wastage rates were calculated for each 6-month interval during the study period. In this model, the pre-intervention slope quantifies the trend for wastage before the intervention. The level change is an estimate of the change in level that can be attributed to the intervention, between the time points immediately before and immediately after the intervention, and accounting for the pre-intervention trend. The post intervention slope quantifies the change after the intervention. We did not perform any adjustments for potential seasonal variance. Units of blood components transfused per patient were summarised using medians (inter-quartile range) and the difference in units of blood transfused per patient between the two periods compared using Wilcoxon Rank Sum test. A *p*-value of < 0.05 was defined to be statistically significant. All analyses were performed using Stata v 18.0 (Statacorp, College Station, Texas, USA).

### Ethics and consent

The study protocol was reviewed and approved by The Hospital Research & Ethics Committee. The requirement to seek informed consent from patients was waived.

## Results

There were 1013 patients in the pre-intervention period and 1175 patients in the post-intervention period who had emergency blood component transfusions in the ED. Baseline characteristics of patients are summarised in Table 1. In the post-intervention period, patients were older; however, the difference was not considered to be clinically significant. There were no statistically significant differences among other measured baseline characteristics.

**Table 1:** Comparison of patient characteristics

	Pre-intervention (*n* = 1013)	Post-intervention (*n* = 1175)	*p*-value
Age in years- mean (SD)	59.9 (SD 21.7)	62.8 (SD 21.2)	0.017
Age in years; categories			0.005
- 18–30 - 31–50 - 51–65 - > 65	139 (13.7%) 201 (19.8%) 214 (21.1%) 459 (45.3%)	117 (10.0%) 213 (18.1%) 234 (19.9%) 611 (52.0%)	
Sex*			0.11
- Female - Male	348 (34.3%) 665 (56.7%)	442 (37.6%) 732 (62.3%)	
Indication for transfusion^†^			0.08
- Anaemia - Trauma - Gastrointestinal bleed - Other	73 (10.4%) 275 (39.1%) 63 (9.0%) 292 (41.5%)	90 (7.9%) 449 (39.5%) 136 (12.0%) 461 (40.6%)	
Initial Glasgow Coma Scale			0.12
- 3–8 - 9–12 - 13–15	146 (14.4%) 29 (2.9%) 838 (82.7%)	135 (11.5%) 33 (2.8%) 1007 (85.7%)	
Initial systolic blood pressure in mmHg; mean (SD)	122.7 (SD 28.6)	123.9 (SD 58.5)	0.80
Initial heart rate in beats/min; mean (SD)	92.0 (SD 21.6)	91.9 (SD 22.8)	0.92
Initial respiratory rate in breaths/min; mean (SD)	19.1 (SD 5.3)	19.0 (SD 4.9)	0.72
Transfusion of > 4 units of red cells	197 (19.5%)	208 (17.7%)	0.30

* Missing data for 1 patient; ^†^Unknown for 349 patients

There were 6619 units of blood components transfused in the pre-intervention period and 6575 in the post-intervention period. Overall wastage rates are summarised in Table 2. There was a significant reduction in wastage rates of blood components associated with the intervention (1.6% versus 1.1%, *p* = 0.008). When sub-grouped to the different types of blood components, there was a significant reduction in red cell wastage (1.7% vs. 1.0%; *p* = 0.005).

**Table 2:** Wastage and transfusion volumes

	Pre-intervention	Post-intervention	*p*-value
All blood components (total units)	6619	6575	
- Transfusion per patient; median; IQR	3 (2–6)	2 (2–6)	0.002
- Wastage	109 (1.6%)	73 (1.1%)	0.008
Red blood cells (total units)	3661	3966	
- Transfusion per patient; median; IQR*	2 (2–4)	2 (2–4)	< 0.001
- Wastage	64 (1.7%)	40 (1.0%)	0.005
Fresh frozen plasma (total units)	1544	1576	
- Transfusion per patient; median; IQR*	4 (2–6)	3 (2–5)	0.04
- Wastage	21 (1.4%)	19 (1.2%)	0.70
Cryoprecipitate (total units)	674	430	
- Transfusion per patient; median; IQR*	10 (5–12)	5 (5–10)	0.008
- Wastage	20 (3.0%)	13 (3.0%)	0.96
Platelets (total units)	740	603	
- Transfusion per patient; median; IQR*	2 (2–4)	2 (2–4)	0.75
- Wastage	4 (0.5%)	1 (0.2%)	0.51

*Among patients transfused the blood component

**Fig. 1:**
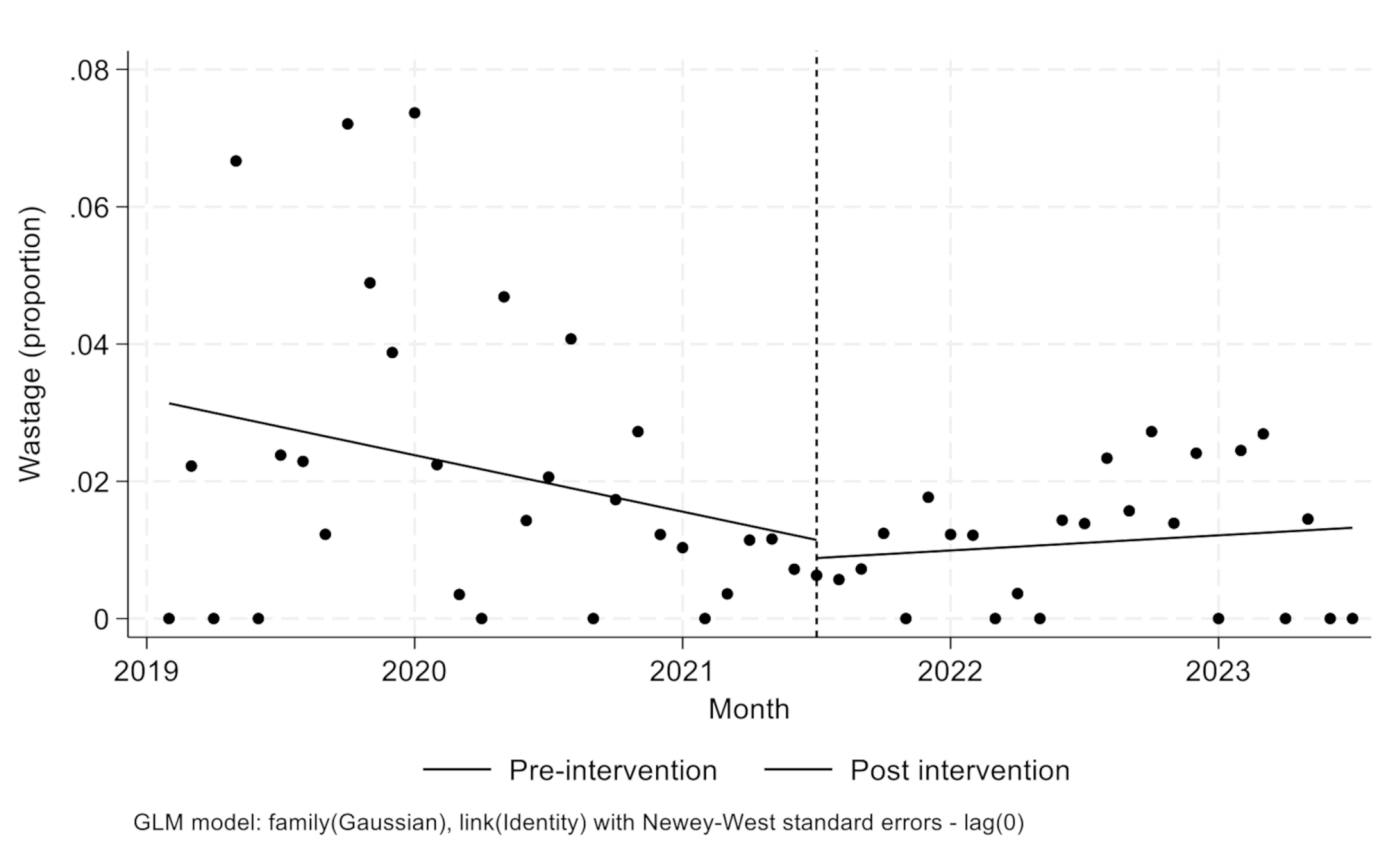
Change in wastage rates over time

Estimates from the interrupted time-series analyses are displayed in Fig. 1. During the pre-intervention period, the coefficient (slope) for wastage was: -0.001; 95%CI: -0.002 to 0.0002; *p* = 0.13). Immediately following the intervention, the coefficient for change was − 0.003; 95%CI: -0.014 to 0.009; *p* = 0.65), while after the intervention, the co-efficient for change was 0.0009; 95%CI: -0.0002 to 0.002; *p* = 0.10). Following the intervention, there were lower numbers of units of red blood cell, fresh frozen plasma and cryoprecipitate transfused per patient (Table 2).

## Discussion

The introduction of a dedicated transfusion team, with the combination all four components of the intervention, was associated with significantly reduced wastage of blood components, specifically for red cells. The volume of blood components transfused was lower after the intervention. Interrupted time-series analysis suggested an immediate reduction of in wastage rates after the intervention, but potential worsening in wastage rates over time. These results support the introduction of dedicated transfusion personnel as part of resuscitation teams but highlight the importance of ongoing reinforcement to maintain the quality of transfusion practices.

The pre-intervention wastage rates in this study were low at 1.6%. The absolute reduction in wastage was 0.5% of all transfusions, this equates to 36 units of blood components in the post intervention period in this single centre. In a report from trauma centres in the USA, with wastage rates up to 9% were reported [9]. Khan, et al., assessed the change in the major haemorrhage protocol at the Royal London Hospital, with the new protocol incorporating a nominated member of the trauma team to communicate with the blood bank on a dedicated phone [10]. Wastage of blood components was 5.5% pre-intervention and 5.6% post-intervention. At the Tampa General Hospital, Florida, the intervention to reduce wastage involved digital timelines to track blood component issued, transfused, returned, and discarded. This was augmented with education of trauma teams and augmented communication between specialty units. Wastage rates of 8% were reported with some indications of improvement after the intervention [11]. The effect of such quality improvement initiatives are therefore more prominent when the pre-intervention wastage rates are higher.

In this study, we did not assess potentially avoidable transfusions. The definitions of potentially avoidable transfusions are variable and challenging to define retrospectively. An overall reduction in blood component use, with similar baseline characteristics, however, provided indirect evidence towards more precise transfusion practice associated with the change. However, it is acknowledged that the transfusion team’s role was operational, and the observed reductions in transfusion could be an association only. Allocation of decision-making capability to this team, with appropriate training, may realise further benefits of dedicated transfusion teams.

While an overall reduction in wastage between the two periods, the interrupted time-series analysis did not demonstrate significant changes in any of its three components. This is likely a reflection of the low baseline wastage rates. It is also possible that reductions in wastage were trending lower prior to the intervention. However, visually, the variations in wastage, with some months of high wastage rates appears to have been mitigated after the intervention. Regardless, a longer time, and further assessment after introduction to other centres is required. We did not observe changes to wastage rates in plasma and cryoprecipitate. These components present targets to refine the intervention, and for further education of transfusion teams.

The potential enabler of the effect was most likely to be a reduction in cognitive load of the team leader, and allocation of staff towards the specific task of transfusion. In the first 30 min of trauma reception and resuscitation, a critical decision is required every 72 s [12]. Without consistent oversight of a team leader, transfusion processes may be delayed, and the existence of blood components at room temperature for extended periods ignored, in favour of other life-saving interventions. As transfusion practices usually follow a protocol, allocation of this process to other clinicians appears justified. This is expected to allow the resuscitation team leader to focus on identifying and managing the cause of bleeding and managing other injuries or medical conditions, with the sole transfusion decision on their behalf being the initiation and cessation of the major haemorrhage protocol. The concept of dedicated transfusion teams within a resuscitation team has been previously proposed [13]. However, it does require the availability of staff that may not always be available, particularly in smaller centres, after-hours and in austere settings.

This study is limited in being a retrospective observational study and therefore cannot imply causation. While the indications for transfusion, vital signs and massive transfusion rates were similar between the two time periods, there are potential unmeasured confounders such as the volume of blood loss and perfusion status. With a low baseline rate of wastage, statistically significant changes in each step of the interrupted time-series analysis could not be demonstrated. An accurate cost-effectiveness analysis of the intervention is possible through quantifying staff-time costs but was outside the scope of this research. Finally, while it is postulated that a transfusion team would have resulted in a reduction in critical decisions that the team leader had to make, this study design did not permit measurement of such decision time-points.

Integration of dedicated transfusion teams into video-assisted resuscitation algorithms is one strategy for consolidation of this practice. There were some suggestions of decay in effect in the post-intervention period. An essential component of the intervention is maintaining the tasks of the transfusion team for transfusion processes only. However, over time, there may be a tendency for the staff to take on other tasks during resuscitation. Ongoing surveillance of the principles of the transfusion team, with enforcement by the resuscitation team leader at the outset of every resuscitation, incorporation of the role of the transfusion team in trauma team checklists and time-outs are other strategies to ensure compliance and effectiveness [14]. 

## Conclusions

The introduction of dedicated transfusion teams during emergency use of blood were associated with lower wastage and lower transfusion volumes. Transfusion teams, rather than the resuscitation team leader, may be well-placed to deliver blood components for transfusion, and to monitor cold chains to reduce wastage. Such an organisational change in trauma teams may lead to more precise management of critical bleeding and reduce wastage and avoidable transfusions, while enabling cognitive capacity for the team leader to focus on identifying and controlling haemorrhage.

## Data Availability

Data can be made available upon reasonable request to the corresponding author, and subject to ethics committee review. Due to the sensitive nature of the dataset, it is not publicly available.

## Abbreviations

CI: Confidence interval
IQR: Interquartile range
mmHg: Millimetres of mercury
SD: Standard deviation
